# Health Systems Approaches to Preventing Chronic Disease: New Partners, New Tools, and New Strategies

**DOI:** 10.5888/pcd16.190248

**Published:** 2019-10-03

**Authors:** J. Lloyd Michener, Peter Briss

**Affiliations:** 1 Department of Family Medicine and Community Health, Duke University School of Medicine, Durham, North Carolina; 2 National Center for Chronic Disease Prevention and Health Promotion, Centers for Disease Control and Prevention, Atlanta, Georgia

The shift from acute to chronic illness as the major source of premature death in the United States and recent developments in health care, such as payments based on results rather than volume alone, are driving fundamental changes in public health and health care. Chronic diseases account for the bulk of morbidity, mortality, and health care costs in the United States. Risk factors for chronic illness are multiple and interrelated; have roots in individuals, families, and communities; and require coordinated strategies across multiple levels and sectors for improvement. These issues are driving substantial change in how health care systems, public health, and other sectors are addressing the chronic disease epidemic. Evolving approaches include coordinating care for people with complex illness; extending the scope of health care systems to new settings; addressing health behaviors and social determinants of health in health care settings and in partnerships with community organizations; using social media to quickly test and disseminate health messages; providing financial incentives and feedback to motivate behavior; and building larger partnerships between public health, health care, and other sectors. Although there are no best practices yet, there are “better practices.”

This *Preventing Chronic Disease* collection highlights some of these evolving practices, drawing from a diverse set of health care systems and public health agencies that submitted articles in response to a call for papers in June 2018. The accepted articles document new approaches for improving systems and addressing upstream causes, intriguing early findings of changes in behavior and outcomes, and changes in workflows that can ease implementation and sustain the improvements.

Health care providers and systems, especially those in primary care, generated multiple examples of systems of care innovations, such as coordinating care for those with HIV infection (1), expanding screening for colorectal cancer through a Medicaid accountable care organization primary care learning collaborative (2), and linking primary care patients with farmers markets (3). A partnership between the American Medical Association and the YMCA, a Center for Medicare and Medicaid Innovation demonstration project, tested increased screening, testing, and referral of Medicare patients with prediabetes seen in primary care practices in 17 US communities to diabetes prevention programs at local YMCAs, supported by a toolkit of workflows and process maps (4). The team was able to achieve a 19% enrollment rate, noting higher referral rates for practices that used a prediabetes registry — an emerging better practice.

Collaborative efforts can be even more powerful when they expand to include the community, as Hearts of Sonoma County demonstrated with its multi-stakeholder campaign to reduce hypertension across its community (5). Similarly, a partnership between the Washington State Department of Health, public and private health care systems, other community organizations, and a supermarket chain launched a fruit and vegetable voucher program. The redemption rate was 54%, and 88% of those surveyed reported an increase in fresh fruit and vegetable consumption (6). People can change their behaviors — but doing so may require that clinicians and public health practitioners first change how we work together and include partners (such as grocery stores, in this case) that traditionally have been seen as outside the scope of influence of either public health or health care.

A substantial challenge in these partnerships is the need for collaborative planning and action, especially given that public health and health care have little history of working together in sustained, coordinated ways. But growing rates of chronic disease, funding challenges within public health, and the shift in health care reimbursement from models based on volume to new models based on value have provided incentives for health care to move upstream. This has also created an opportunity to establish local coalitions that include public health and health care and other critical groups with a common goal of improving health in their communities. Such coalitions are growing rapidly; one recent assessment (7) found almost 600 partnerships for health under way across the United States, with a range of areas of members, focus and structure, and key roles for public health in convening groups and in sharing results and lessons learned (8).

But partnerships do not just happen, nor does having partners guarantee success. The Hearts of Sonoma County program (5) found that the key elements were starting small, building trust, having a framework, and providing long-term backbone support. The larger Heart Disease and Stroke Prevention Learning Collaborative, a cooperative agreement between the Association of State and Territorial Health Officials and the Centers for Disease Control and Prevention (CDC) (9), used a systems change framework, teams, and expanded self-management options and found them effective, including in rural settings. CDC supported state collaboratives with child care resource and referral networks in 10 states (10), tested methods of supporting adoption of best practices, and found improvements overall, suggesting that it may be the partnerships themselves, in addition to the programs, that make a difference. Over time, linkages can become extensive and strong, as has been the case in Nebraska (11), requiring effective management and continued attention to ensure community priorities remain paramount.

Picking effective methods helps, too. Clinical groups can provide needed services outside clinic walls, as is well demonstrated by the success of mobile mammography units in reaching underserved women (12). Public health (among others) can use social media to test the effectiveness of different messages at low cost, permitting highly tailored health messaging (13). Working together, public health, health care, and other agencies can use vouchers and feedback to help achieve public health goals, such as reducing radon exposure (14).

The evaluation methods reported are striking for the predominance of mixed methods; the use of diverse data sources, including commercial health claims data sets (15,16), electronic health records (4), geotags (16), and new measures such as Facebook click-through rates (13); the use of well-established frameworks such as RE-AIM (Reach, Effectiveness, Adoption, Implementation, Maintenance) (4); and for their high level of sophistication (10). Although it is possible to use simple evaluation methods when assessing the value of single components of a larger program (such as the comparative value of different social media methods [13]), evaluation of large, complex programs requires considerable expertise, planning time, and funding.

These are early reports, and much work remains. Most of the reports address system changes and upstream causes one person at a time, which is welcome progress but far from addressing root causes. Many of these reports focus on disparities and underserved populations, but more work is needed in this area so that we learn how to effectively partner with the wide range of people, cultures, and settings across our states and communities. Despite the near ubiquity of data on health outcomes and risk factors by county and census tract, such as County Health Rankings (17) and 500 Cities (18), few use such data to target their efforts. Rates of use of preventive services remain low, requiring continuing experiments to find what works in what setting (19) as well as what issues (such as cost to participants) must be considered in planning (15). Expanding partnerships to include businesses, elected officials, and other actors can help reframe perspectives on cost and benefit, as stakeholders learn that the health of the communities is of value far beyond costs and outcomes of health care (16).

What does that mean for public health? First, public health cannot reduce chronic disease rates alone, and neither can health care. Each has an essential and complementary role, with public health engaged in establishing and supporting partnerships and health care contributing its resources, including data, and powerful voice in advocacy. Second, voices of the community must be present and heard in all their diversity, especially in communities in which trust in government, health care, and other sectors has been lost. Third, partnerships are hard work and require infrastructure and support (which need not always come from public health funds!). Last is the need for a sense of both humility and excitement as we learn to work together to help free our communities of the burdens of chronic disease. We hope the articles in this special collection mark both some of the early successes and lessons learned in our journey together.
